# Are investments in malaria control saving the lives of children? Challenges in using all-cause child mortality for measuring the impact of malaria control programs

**DOI:** 10.1186/1475-2875-13-S1-O27

**Published:** 2014-09-22

**Authors:** Thomas Eisele

**Affiliations:** 1 Tulane School of Public Health and Tropical Medicine, New Orleans, USA

## 

As global investments to combat malaria have reached over 1.8 billion per year, there is a strong desire by governments and donors to measure the number of deaths prevented from these investments. Much of this funding has gone to African countries where malaria burden is highest, but robust health information systems and vital registration are weakest, which precludes measuring changes in malaria deaths directly. Because malaria accounts for a large proportion of deaths in Africa (15%), assessing trends in all-cause child mortality (ACCM) has become a key indicator for measuring program impact. The objectives of this talk will be to provide a comparison of common epidemiological approaches used for evaluating the impact of malaria control programs on ACCM in the African context. Three examples of impact evaluation approached using ACCM as the primary outcome will be discussed: 1) comparison of ACCM measured by household surveys before and after malaria program introduction or scale-up (aka ‘trends over time’); 2) assessment of the association between exposure to malaria control interventions (e.g. insecticide-treated mosquito nets- ITNs) and ACCM using cross-sectional data; and 3) an analytic frameworks that allow a dose-response relationships to be estimated between changes in sub-national (district) malaria program intensity and ACCM. In doing so, an attempt will be made to discuss the key challenges of using these methods, as well as specific recommendations for their improvement.

